# The Future of Medicine: Frontiers in Integrative Health and Medicine

**DOI:** 10.3390/medicina57121303

**Published:** 2021-11-28

**Authors:** Mahadevan Seetharaman, Geetha Krishnan, Robert H. Schneider

**Affiliations:** 1 School of Health Sciences, Institute of Transdisciplinary Health Sciences and Technology, Bengaluru 560064, India; 2 AYUSH Global, Sugar Land, TX 77479, USA; 3 Traditional, Complementary and Integrative Medicine Unit, Service Delivery and Safety Department, World Health Organization, 1211 Geneva, Switzerland; drgk2000@gmail.com; 4 College of Integrative Medicine, Maharishi International University, Fairfield, IA 52556, USA

## 1. Background

Despite advances in modern medicine, contemporary society has experienced a series of epidemics and pandemics of noncommunicable, chronic diseases and communicable, infectious diseases [1,2]. These public health crises are related, at least in part, to behavior and lifestyle [3]. There is increasing interest and growing evidence that the integration of conventional medicine with traditional, complementary, and alternative medicine (TCAM) may be useful for the prevention and treatment of communicable and chronic diseases related to behavior and lifestyle [4,5,6]. The World Health Organization (WHO) has been developing standards and documentation for the state and practice of TCAM [7]. The aim of this special issue of *Medicina* on “The Future of Medicine: Frontiers in Integrative Health and Medicine” was to explore the scientific evidence for these approaches of integrative medicine, which hold promise for the future of healthcare and medicine.

According to the Consortium of Academic Health Centers for Integrative Medicine and Health, integrative medicine is defined as the practice of medicine that reaffirms the importance of the relationship between practitioner and patient, focuses on the whole person, is supported by evidence, utilized all appropriate therapeutic and lifestyle approaches, healthcare professionals and disciplines to achieve optimal health and healing [8,9,10]. TCAM includes modalities such as Ayurveda, Yoga, traditional Chinese medicine, other traditional systems of medicine, meditation, herbal medicines, nutritional supplements, movement therapies, and other mind-body practices. The World Health Organization (WHO) now refers to this set as Traditional, Complementary and Integrative Medicine (TCIM) [11].

There has been a surge in the public interest and the use of TCIM globally. Nearly 50% of the population in developed nations (United States, 42%; Australia, 48%; France, 49%; Canada, 70%), and similar or greater numbers in developing countries (India, 70%; China, 40%; Chile, 71%; Colombia, 40%; up to 80% in Africa) use some form of TCIM [12]. The World Health Organization and governments of several countries have established agencies to support research and practical utilization of TCIM [13].

**Figure 1:**
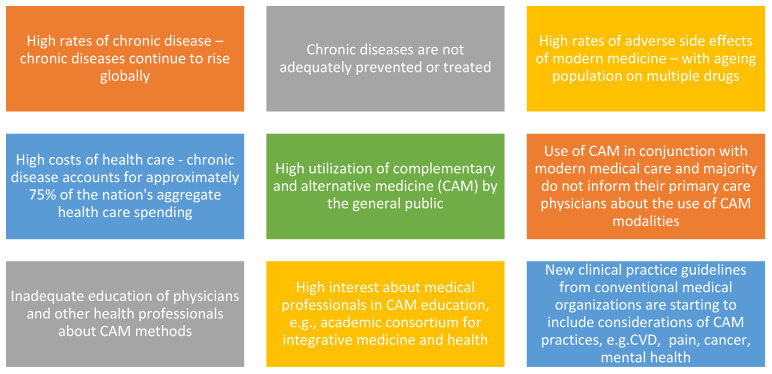
Trends driving integration of TCIM in contemporary healthcare. TCIM: Traditional, Complementary and Integrative Medicine; CVD: cardiovascular disease.

The trends driving integration of TCIM in contemporary healthcare are presented in Figure 1. According to the WHO, non-communicable or chronic diseases continue to rise globally [14,15]. These diseases are not adequately prevented or treated by modern medicine and are often accompanied by high rates of adverse side effects. With an ageing population using polypharmacy, adverse events are also increasing [16] The costs of modern healthcare are high [17], with chronic disease accounting for approximately 75% of the US aggregate health care spending according to the Centers for Disease Control and Prevention (CDC) [18,19,20,21]. The trends also show high utilization of complementary and alternative medicine (CAM) amongst the public with most patients using CAM in conjunction with modern medical care and majority of them do not inform their primary care physicians about the use of CAM modalities [4]. There is inadequate education of physicians and other health professionals about CAM methods, and high interest amongst medical professionals in CAM education according to the Academic Consortium for Integrative Medicine and Health [22]. New clinical practice guidelines from conventional medical organizations are starting to include considerations of CAM practices, e.g., in cardiovascular health [23], cancer [24], pain [25] and mental health [26].

At present, the growing use of integrative medicine has stimulated the scientific community, national and global policy organizations to investigate the clinical efficacy, mechanisms of action, healthcare costs, standards, protocols and policy implications of these therapies [27,28,29]. However, with notable exceptions, much of this effort has been on quality, safety, and efficacy of TCIM products mainly due to the potential for exploitation in drug discovery and development [30].

## 2. Current Contributions

The goal of this special issue of *Medicina* was to address these gaps in collective understanding and to promote the integration of TCIM into contemporary medicine and public health. This collection of articles will stimulate further research and support healthcare professionals, consumers, and policy makers to make informed decisions about the utilization of evidence-based integrative medicine for the future of healthcare.

There are several critical observations and implications on the management of communicable and non-communicable diseases based on this series of papers. Using microarray experiments, Wenuganen et al. studied the effects of Transcendental Meditation (TM) practice on the expression of 200 genes [31]. Compared to control group, 49 inflammation related genes were downregulated, while genes associated with antiviral and antibody components of the defense response were upregulated. Meditation might have an important role to play in the management of chronic diseases, including anxiety, post-traumatic stress disorder (PTSD), cardiovascular disease (CVD) and communicable diseases. As meditation practices have become more common place, Travis presents three strategies to organize and study brain patterns during meditation practice [32].

We are still in the midst of the SARS-CoV-2 pandemic and the scientific and medical community is looking for novel therapeutic and preventive anti-viral candidates. Ali et al. (2021) highlight natural medicines that could be used against SARS-CoV-2 including plants like *Glycyrrhiza glabra (licorice)*, *Alnus japonica (alder)*, *Allium sativum (garlic)*, *Houttuynia cordata*, *Lycoris radiata*, *Tinospora cordifolia (guduchi)* and *Vitex trifolia (nirgundi)* [33]. Vitamins such as A, B2, B3, B-12, C, D and E, and minerals such as Zinc may have a possible role in SARS-CoV-2 prevention by enhancing immunity.

Kshirsagar and Rao (2021) present recent studies that have investigated derivatives from the plant Artemesia that is widely used in Ayurveda and traditional Chinese medicine (TCM) for its antiviral, antifungal, antimicrobial, insecticidal, hepatoprotective and neuroprotective properties [34]. The notable phytochemical Artimesinin from Artemesia has shown not only to have potent antiviral actions but also utility against the severe acute respiratory syndrome coronavirus 2 (SARS-CoV-2). Youyou Tu was awarded the Nobel Prize in 2015 for the discovery of antimalarial properties of Artemisinin [35], which holds promise for anti-viral and anti-inflammatory drug discovery from traditional plant sources.

The traditional healthcare system of India, Ayurveda typically recommends changes in diet, lifestyle, behavior, digestion, stress, and environmental factors for prevention and treatment. These are factors that cause epigenetic changes and affect gene expression. Sharma and Wallace propose that epigenetics is an important mechanism of Ayurveda and studying the effects of Ayurveda-based modalities on gene expression will increase understanding between Ayurveda and modern medical science [36]. Wallace reviews and provides an update on the developments in Ayurgenomics, the field that integrates concepts in Ayurveda, such as Prakriti, with modern genetics research. He suggests that the *Tridhosha* theory of Ayurveda (the combination of three physiological modulators—Vata, Pitta and Kapha) correlates with the expression of specific genes and physiological characteristics [37].

According to Ayurveda, the gut has an important link to the health of an individual. Diet and digestion influence the composition of the gut microbiome. Ayurveda has been focusing on the role of digestion in health and disease for millennia. The author presents the connection between the gut microbiome and various prevention and therapeutic approaches of Ayurveda [38]. This has major implications in the field of integrative Ayurveda that combines the best practices of Ayurveda with modern medicine.

Arteaga-Badillo et al. (2021) present new integrative treatment strategies for the management of asthma [39]. Scientific evidence suggests that diet including fruits, vegetables, seeds, whole cereals, consumption of vitamins A, C, and E and the use of plants and natural extracts (phytotherapy) may help relieve symptoms of asthma. In particular, plants such as *Glycyrrhiza uralensis*, *Angelica sinensis*, *Pinellia ternata*, *Astragalus membranaceus*, *Helichrysum stoechas*, *Eucalyptus globulus*, *Rosmarinus officinalis*, *Zingiber officinale*, *Inula helenium* and *Allium sativum* L., have shown significant benefits.

A systematic review and meta-analysis of Ayurveda based herbal preparations for hypercholesterolemia is presented by Gyawali et al. (2021). These authors found that three Ayurvedic herbs—garlic, guggul and black cumin, were safe and effective in reducing cholesterol biomarkers. Several other Ayurvedic interventions that include the herbs, holy basil, ginger, fenugreek, arjuna and Indian gooseberry may also ameliorate hypercholesterolemia [40].

Megas et al. hypothesize that conventional therapy integrated with Anthroposophic therapies may be potent and beneficial for plastic surgery patients. In this approach, not only functional and physical approaches are stressed, but also mental state, creativity, and self-determination. Complementary approaches include Anthroposophical massage (rhythmical massage and streaming massage), breathing therapy, ergotherapy, eurythmy therapy, hyperthermia, painting therapy, clay modelling therapy, music therapy, physiotherapy, and psychotherapy. Along with these mind body practices; natural products are incorporated [41].

Steinbaum argues in her perspective article that women have been proportionately affected by the COVID-19 pandemic. Of the 5 million women-run businesses, 25% of them closed within two months of the pandemic outbreak. These stressors impact psychological and physical health. Stress and lack of self-care increases chances of heart disease, the number one killer in women. Stress impacts behavior, resulting in overeating, sedentary behavior, poor diets, increased alcohol intake and lifestyle, all preventable risk factors for heart disease [42].

Jonas and Rosenbaum shared their perspective on the need for standardization of whole-person models and research using whole systems approaches [43]. Whole person medicine includes dimensions of health that consider behavior and lifestyle, social and emotional dimensions, and an individual’s mental and spiritual dimensions. The authors present the status of current evidence on holistic models for improved health outcomes, patient satisfaction, health care costs and clinical experience.

Purushotham and Hankey [44] conducted a detailed comparison of the dietary intake of food groups in two cohort studies of diet and stroke that had disparate results. They applied the nutritional principles of Ayurveda to suggest how these apparently contradictory results may be explained. The authors propose that traditional systems of medicine, such as Ayurveda possess clinically relevant knowledge of the effects of food on physiology and health.

The TCIM modalities reported in this special issue may be applied to the COVID-19 pandemic, prevention of future pandemics, and to address the on-going epidemic of non-communicable, lifestyle-related diseases. This collection of articles stimulates further research in integrative medicine and supports healthcare professionals to make informed decisions about the practice of evidence-based integrative medicine in contemporary healthcare settings around the world.

## 3. Post-COVID-19 Era: An Integrative Vision of Healthcare—Public Health and Policy Recommendations

At the time of writing, COVID-19 infections and death continue in pandemic proportions [45]. Clinical management, research and therapeutic strategies for COVID-19 have focused on either tackling the virus directly or immunize against it. Over the past century, society experienced a series of pandemics or epidemics, notably polio, smallpox, cholera, plague, dengue, AIDS, West Nile, tuberculosis, severe acute respiratory syndrome (SARS) and now COVID-19 [1,46]. On the contrary, Ayurveda, amongst the oldest and most widely practiced traditional healthcare systems, recommends preventive measures for improving immunity through healthy lifestyle in addition to acute therapy when indicated. Ancient Ayurveda texts, notably *Charaka Samhita* discuss epidemic management and immunity enhancement to prevent disease, arrest its progression and to maintain health on the individual and public health scales [47]. The Ayurvedic texts explains the onset of pandemics when there is collective human stress the disrupts endogenous preventive capacities. The concept of building and maintaining balanced functioning of the mind and body to cope with internal and external stressors, including infectious microorganisms, is the fundamental strategy of Ayurveda [48]. Many of these preventive principles have garnered modern scientific evidence which then supports the proposal that they are physiologically applicable across cultures and geography [48,49,50].

An integrative approach to healthcare that focuses on prevention and immunity building is imperative to preventive future pandemics. Such an approach to healthcare would involve modern medicine education and practice incorporating traditional, complementary, and alternative medical approaches including Ayurveda, Yoga, Chinese medicine, and other traditional systems of medicine. The development of an integrative curriculum will lead to a more complete understanding of optimal health and wellness. In modern scientific language this is termed, systems medicine which encompasses the individual, family, community, and the environment [51,52]. Ayurveda describes this systems approach to health care in the concept of ‘*Swasthya*’ or wholeness. That is, optimal health and well-being are based on inner wholeness together with a balance of mind, body, social and physical environments [49,52,53]. This would require a revision of health care for the 21st century that shifts the paradigm from a standardized approach to personalized prevention and treatment, from a short-term to long-term sustainable intervention, from single molecular targets to an integrated system of networks and, from treatment with adverse effects to prevention and holistic health promotion.

In a way, the COVID-19 pandemic has been an eye opener for the scientific community and policymakers alike. It has shown to us that the resources and knowledge, the skills and human capital, the medicines, and the infrastructure, all fall behind expectations when faced with a pandemic of this stature [54]. It calls out at us that the current preparedness to achieve the sustainable development goals (SDG) of the United Nations is a mirage and unachievable, unless deliberate drastic, and systemic changes are brought about in their approach by communities and nations, in its pursuit [55]. It is evident that unless all resources available to human societies are utilised in the most effective an appropriate manner, we might be found formulating an SDG 2050, in the year 2030 without much change of its expected outcomes. Integrative medicine therefore is not a possibility but and a necessity. Human societies, whether technologically and financially advanced or deprived, will have to find their own integrative models in public health to achieve their expectations in health outcomes by 2030. In Africa, for example, an integrative model might look at skilling traditional practitioners to identify diseases, create knowledge base of available medicinal interventions, support evidence generation, provide guidelines for specific clinical interventions which are safe and effective, and develop and establish clear referral models [56,57,58,59]. In India, the home of Ayurveda, the contemporary health care system might utilize the country’s range of traditional modalities (AYUSH) in prevention and early management of diseases, and health management of categorised population groups such as the aged, women and children, healthy adults, palliative care etc [60]. In the United States, Germany, or Canada it might evolve to support specific public health initiatives in cancer care, pain management, palliative care etc. It could also extend to clinical conditions such as sleep medicine and support specific neurodegenerative conditions. Emerging areas such as wellness or mental health promotion, will progress faster, when TCIM knowledge gets integrated into research, practice, education, and policy. Whichever may be the course taken by contemporary society, integrative medicine is here to stay and nations who ignore its precious resources would do so at their own economic and health costs.

## Data Availability

Not applicable.
